# Hospital and Institutional News

**Published:** 1914-12-05

**Authors:** 


### HOSPITAL AND INSTITUTIONAL NEWS.

### THE KING IN HOSPITALS IN FRANCE.

The arrival of the King in France has not only revived a historical precedent in the history of our monarchs and their troops in the field; it has also shown by the first visit paid by him his well-known interest in hospitals. The "town in France" where he arrived possesses a hospital in which he at once inspected the men's wards, speaking to some of the wounded soldiers, including a German officer, with whom he conversed in his own tongue. The King also pointed out the need for supplying the German wounded with literature owing to the deprivation which they suffer from being largely unable to converse with other wounded men. The Prince of Wales accompanied His Majesty, who spoke to each patient in the officers' wards. After visiting another hospital for British officers, where he again spoke to every patient and praised the medical and the nursing staff, the King also paid a visit to the Indian hospital, where he remained for three-quarters of an hour. Such an event will be a unique memory for the men, and the sepoys were delighted not merely at his inquiries concerning their wounds, but at the knowledge which the King displayed of the actions in which they had been engaged, including the names of the regiments. The patience of his wounded Indian troops under pain in hospital much impressed the King, who inspected every ward, the theatre, *x*-ray room, the thresh, the Hindu and Mohammedan kitchens, and even the laundry. This inspection will bring home to English readers, as our second leading article notes in the case of Poor-Law infirmaries at home where Indian wounded are being treated, how complex are the institutional problems created by the caste system and religious observances of our Indian fellow-subjects. The convalescent camp was also visited, where the wounded Indians in tents, under similar conditions to those of a cantonment, took advantage of their opportunity to give His Majesty a tribute of applause which we may be sure both he and they will long remember. For the visit is truly historic both among royal visits to hospitals and in the annals of our English and Indian armies in the field.

### HOSPITAL SECRETARY KILLED IN ACTION.

We learn with great regret that Sergeant A. E. Thomas, secretary of the Hampstead General Hospital, who was mobilised with the Honourable Artillery Company on the outbreak of war, has been killed in action on November 25. Sergeant Thomas was in the trenches hard at work when a bullet penetrated his neck, and he died only four minutes later. It is, indeed, believed by those most likely to know that his keenness was the cause of his undoing, for he was so anxious to direct the firing to the best advantage that it is probably due to his insufficient caution in movement which made him a target for the enemy. As his many friends in the hospital world know, he joined the Honourable Artillery Company in 1908, and made his work for the Company the second interest of his life. He was as popular in the regiment as outside, and keenness and capacity distinguished him here as in hospital life. The H.A.C., which was originally founded by Royal Charter, under which the commissions were signed by the Sovereign, is now on precisely the same footing as the other regiments under the Territorial Force Act. But we in the hospital world will rejoice chiefly to recall that Sergeant A. E. Thomas was one of its able workers, and as hon. treasurer of the Hospital Officers' Association he was known to a wide circle. In connection with his death it is somewhat pathetic that, in the letter of his which appeared in the November number of the Association's journal, after describing the local hospital arrangements in the town where his—No. 1—company was stationed, he wrote as his concluding words: "We are now under orders to move on, and we shall probably be seeing something more interesting soon." This proved to be the trenches and the firing line, with his death as the sad, but worthy, climax.

### ENGLAND'S FIRST HOSPITAL WARRIOR.

Mr. Thomas's career as a hospital secretary, we may note, was in some respects curious. After having been secretary of the Gravesend Hospital he took what many people would have thought the impolitic step of becoming an assistant secretary, at the Royal Free Hospital. There he remained for years, until in May 1910 he was appointed secretary to the Hampstead General Hospital. Two years later, when a vacancy occurred in the secretaryship of the Royal Free Hospital, Mr. Reginald Garratt was elected. The opinion was expressed at the time that had Mr. Thomas been assistant at the date of his senior's retirement no other candidate, however strong, would have been likely to be elected. Keen and capable at whatever he put his hand to, Mr. Thomas was an enthusiastic Freemason, being a member of the Nosocomia Lodge for hospital workers, and also of Fitzroy Lodge for members of the Honourable Artillery Company. He was unmarried, but his mother survives him, and to her, as to his many friends, we may respectfully offer our condolences in a bereavement which removes from the hospital world and from the Army an energetic and capable gentleman in the prime of life. Mr. Thomas was one of the men of 1914 who responded to his country's call, and has met his death in her defence. We hope the Royal Free and the Hampstead General Hospitals will each shortly contain a marble tablet to his memory as the first hospital hero to perish in the war for freedom.

### TRADING WITH THE ENEMY?

Through the courage and patriotism of the matron of the Royal Hospital for Incurables, the *Nursing Mirror* is able to print this week a remarkable letter received by her from a firm with a German-sounding name, describing themselves as waste-paper and gutta-percha merchants, import and export, of Finsbury Square. Prefacing her communication with the remark that "It is well known that there is a great shortage of rubber in Germany," the text of the letter reads as follows: "Dear Sir,—We shall be glad if you will let us know whenever you have any waste rubber for disposal, and beg to make formal application to be placed on your list of buyers. We are always open to purchase any quantity and kind of scrapped india-rubber stores, such as water beds and bottles, waterproof sheeting, etc., and would, in case of business, send one of our vans with a representative to take delivery of the goods against cash." The letter, which is signed "S. Schein and Sons," and ends with the words "Attention: The Steward," may or may not emanate from German sources; but since the feeling against trading with the enemy is very strong, and German methods are now much open to suspicion, it was a public-spirited and sensible course to give publicity to this communication. It would be interesting to know how many other hospitals have received a similar request, and what are the views of institutional officers on whether or not this is an invitation for "trading with the enemy."

### THREE D.S.O.s FOR "ATTENDING THE WOUNDED."

The list of fifty-eight officers appointed to be Companions of the Distinguished Service Order, in recognition of their services with the British Expeditionary Force, which was dated from the War Office on Monday, contains the names of the following three members of the Royal Army Medical Corps:—Captain James Stuart Dunne, R.A.M.C., who, in the words of the official statement, "during German attack on night of October 31, near Messines, established a dressing station just behind the trenches, and was the means of saving many lives, he himself going several times into the trenches to attend to wounded men who could not be moved." The second name is that of Captain Patrick Sampson, R.A.M.C., who is said to have "shown frequent and conspicuous gallantry throughout the campaign, especially on October 21 and 22, attending wounded men under very heavy shell fire." The third name, which we are privileged to give, is that of Captain Sidney John Steward, R.A.M.C. (Special Reserve), who is remarked on as one who "went with party of stretcher-bearers across ground swept by rifle and shell fire to Langemarck village and removed eleven wounded men." It is noteworthy that these distinctions have been conferred upon members of the Royal Army Medical Corps in each case for bravery in attending to the wounded—in two instances, we are particularly told, under very heavy fire. It is, therefore, clear that our contributor from the Front with the Corps was fully justified in saying that there was every need for care and common-sense, so great is the inevitable risk of valuable lives in the medical work demanded.

### BOMB-DROPPING ON THE HOSPITAL AT BAILLEUL.

The latest of the, at present, relatively few instances of bombs dropping upon hospitals has been described by an eye-witness, as occurring on November 21 at the hospital at Bailleul. "I heard it fall," says the writer, "and went out to look and saw the German aeroplane sailing away. It fell into a portion of the clearing hospital, about 150 yards from here. The hospital was in a college with a chapel attached to it. The room the bomb fell in was a library, one-storeyed; it came through the roof and ceiling and burst where there were only two sick or wounded and two orderlies. It killed one man and wounded two and made hay of the library. I went and had a look at it. The ceiling was all torn to ribbons; every window near was smashed, including the stained-glass windows of the chapel, which were on a level with the roof of the library." Then, as has been a coincidence several times remarked on by writers on the look-out for facts of this kind, the eye-witness notes that while the "walls had many marks on them," he observed that "a large crucifix on the wall was absolutely untouched." What is even more remarkable is that only two wounded and two orderlies were in the room where the bomb burst at the time.

### THE EMPLOYMENT OF BELGIAN PHARMACISTS.

The question raised in last week's issue of The Hospital as to whether it is feasible or expedient to employ Belgian pharmaceutical refugees in English pharmacies has created some interest. The principle underlying the resolution of the Departmental Committee on Belgian Refugees to the effect that Belgian labour should only be engaged in occupations in which no suitable British labour appears available is, of course, a sound one for general application, and no violation of this principle would necessarily be involved by the employment of Belgian pharmacists; for while it would not be strictly correct to state that no English labour is available in the case of pharmacy, there is undoubtedly room for as many Belgian pharmacists as are likely to seek refuge in this country without the displacement of English assistants. Ever since the Insurance Act came into operation the demand for chemists' assistants has been at least equal to the supply, and the difficulty of replacing assistants has now been accentuated because so many of them have joined the Colours. The employment of Belgian pharmacists would, therefore, be the means of relieving the position, at any rate, to some slight extent. Possibly the number of Belgian pharmacists who would desire to obtain employment in chemists' shops or in hospitals would be fifty or sixty at the most. We note the extraordinarily low salaries at which some are offering their services.

### THE BELGIAN LANGUAGE AND THE SCIENTIFIC STANDARD.

The language difficulty is not so great as would be supposed. Some of the pharmaceutical refugees have at least a bowing acquaintance with the English language, and a few of them speak it quite well; but, what is still more helpful, a considerable number—amounting to at least several hundred—of English pharmacists speak French fluently, having learnt that tongue in Paris, Nice, Cannes, and other parts of France where English assistants are employed for the convenience of English visitors. In the cosmopolitan quarters of London many French assistants are employed at normal times, and at present, while the French-speaking population is above the average, a sprinkling of Belgian assistants would serve a useful purpose. The standard of the pharmaceutical curriculum in Belgium is at least as high as it is in England, and from the scientific point of view the Belgian pharmacist is at least the equal of his English *confrère*. The difference in the strength of the preparations of the two national Pharmacopœias could soon be learnt by a trained pharmacist; and, providing the requirements of our pharmacy law are strictly observed, the employment of Belgian pharmacists does not appear to present objectionable features.

### TWENTY-ONE YEARS AS TREASURER.

Mr. W. Precey has resigned the post of honorary treasurer to the Walsall Hospital on the grounds of ill-health, after having held it for twenty-one years. The growth of work in hospital departments has not spared those responsible for its finances, and the problems of investment and selection of securities, together with the effects of legislation, have made the responsibilities of this office, even in the case of the smaller hospitals, far greater than they were twenty years ago. In his late capacity as manager to the Walsall Branch of the Metropolitan Bank (of England and Wales), Limited, he was called upon to act as treasurer for many charities. He retired from this post some three years ago. Mr. Precey, while believing in conservative finance for hospitals and being averse on principle to overdrafts, will chiefly be remembered for the introduction of the Uniform System of Hospital Accounts in 1907.

### A BANK MANAGER'S WILL.

Mr. James Edward Harris, of Bank House, Penarth, manager of the Cardiff Docks branch of the London and Provincial Bank, Limited, who died on September 15 last, aged sixty-two, left estate of the gross value of £30,193, of which the net personalty has been sworn at £29,319. It is estimated that a sum of not less than £25,000 will be available for charitable purposes, entirely at the discretion of the executors. It should be unnecessary to remind them of the fine work which the Cardiff voluntary hospitals are now doing, and of the claims for support which this active centre has, now that the scheme for giving Cardiff a medical school must add to the prestige of the city and its hospitals.

### IRELAND AND THE EXTENSION OF MEDICAL BENEFIT.

On Friday last week the report of the Committee appointed to advise with regard to the application of the Insurance Act to out-workers in Ireland was published as a White Paper. Its first point is that the omission from the Insurance Act as applied to Ireland of medical benefit militates gravely against the adoption of the scheme. The Committee, therefore, recommends that no change should be made at present in the application of the Act to Irish out-workers, those mainly dependent on their outwork remaining insurable, and the remainder continuing outside the scope of the Act. If, on the other hand, the recommendations of the committee appointed to consider the extension of medical benefit to Ireland are adopted, then all out-workers in the areas to which medical benefit is extended should be brought within the Act immediately, and be eligible for the same benefits as those to which in-workers are entitled. In short, the Committee is of opinion that until medical benefit be extended to Ireland nothing can be done.

### DEATH OF A HOSPITAL SECRETARY.

We regret to announce the death of Mr. Arthur Hulme, who so recently as July retired from the secretaryship of the Queen's Hospital, Birmingham, and has been seriously ill for some time. The funeral took place last week, Mr. Hulme having died on November 21. As we recorded at the time of his resignation, he had been secretary and superintendent of the Queen's Hospital, Birmingham, for upwards of thirty years. Since the city is a keen hospital centre, conspicuous for the solidarity and co-operation of its institutional officers, having for instance, its own Charities' Secretaries' Club, his loss will be felt by all his brother officers. It is a sad coincidence that Birmingham should have lost by death within almost a few days of one another the two administrative heads of its well-known general hospitals. The late Mr. Howard Collins' successor at the General Hospital, Birmingham, has not yet been made known.

### THE PHYSIOLOGY OF SOLDIERS' SONGS.

A correspondent sends us the following: "Speaking at University College, Nottingham, on Saturday, to the members of the Nottingham and East Midlands Branch of the Music Teachers' Association, Mr. Thomas Henderson, Mus. Bac., of Darlington, said he believed that the rate at which a composer breathed had a great influence upon his compositions. Judging from their works Handel and Beethoven respired more slowly than Mendelssohn. Most people breathed about twenty times a minute, and he saw in that a reason why, quite apart from the melody, the most popular music was that in which the rhythm was even and the accent always came on the beat. The song. 'It's a long, long way to Tipperary,' was a case in point. It had been asked why it should be more popular than Elgar's patriotic song, 'Land of Hope and Glory.' The reason was that 'Tipperary' was in agreement with the theory of natural respiration, while Elgar's song was not. The phrases were too long and were apt to exhaust the breath before the end of them was reached." In this connection our readers will recall how the officer of the R.A.M.C. at the Front in his article last week, referring to the sentimentality of the soldier, remarked that "the favourite songs" were not only slushy in sentiment but "sung to dirge-like tunes so slowly that it seems as if the singer were unwilling to part with each note." Are we to draw the conclusion, however, that the "theory of natural respiration" requires sentimentality of the slushiest kind in song and music? Since

Mendelssohn was more sentimental than Beethoven, who had a good deal of rollicking fun in him, slow breathing would seem to be in favour of bracing rhythm and words. Why then, again, should "natural respiration," naturally preferring short phrases, yet love to drag them out as if soldiers preferred a wake to a concert? Perhaps some musician, who is also a doctor (of medicine), will provide an answer to this curious point?

### "TRUTH" CHRISTMAS NUMBER.

For so many years has the *Truth* Toy Fund at Christmas done public service by acting as almoner to all outside the workhouses who are anxious to give the children within them as happy a Christmas as possible, that its illustrated Christmas Number comes home to institutional workers more pleasurably than that of any lay newspaper. Certainly the Christmas Number just issued is a feast of illustration and humour. The war is, of course, the main theme, and the two coloured cartoons by Mr. Stanger Pritchard and Mr. Roland Hill ("Rip") are imaginative pictures of horror and comic effects respectively. The idea of putting the Kaiser in the dock on the charge of hooliganism, before a jury of neutral sovereigns, is characteristically exuberant after "Rip's" manner, and the literary contents, mainly represented by the potted epic in which Jove punishes the Kaiser for threatening his throne, in the classic manner, by driving him mad, makes very good reading. The Kaiser is represented as Super everything, except, which might have formed the epilogue, superannuated; and institutional readers will note that the sharp tongue and curious eye of *Truth* in detecting the bogus exploiters of charity rings quite as merrily, if more genially, in this well-thought-out and most amusingly presented Christmas Number. *Truth*, as a kind of lay almoner throughout the year to charities, will have a cordial welcome in wards and officers' quarters at Christmas-time.

### THE WAR'S EFFECT ON A LONDON EYE HOSPITAL.

The removal of the Central London Ophthalmic Hospital to Judd Street, where its new premises were briefly described at the time, appears, through the war, to have been accompanied by a serious falling off in subscriptions. The committee indeed has felt impelled, owing to this cause, to close some of the beds to prevent them from running into debt. We do not want to add to the hospital's difficulties at a time when all voluntary institutions must stand together, but our views that it is a misguided policy to close wards and beds are well known. Since the administrative expenditure remains practically the same, what advantage is there? and the idea that the public sympathy is stirred by this policy is not borne out by experience. As the hospital now can claim that "everything necessary for the treatment of the eye" is provided by its new and up-to-date equipment, its policy must be to do as much of the work, which the public and the military authorities require to be done, as possible, in so far as that work comes within its scope. Are there no would-be recruits who could pass the eye-tests with the hospital's aid? Are there no men in Kitchener's Army who, after enlisting, require treatment? Until these questions have been answered in the negative, which a vigorous forward policy, we believe, would render unlikely, the Central London Ophthalmic Hospital should have no need to practise the false economy of closing beds, and would, we are convinced, by informing the public of its work for the nation, be roping in new and increased subscriptions. What does Mr. Druce, the secretary, and his committee think on this head?

### INCREASE OF SALARIES DURING THE WAR.

There has been a growing feeling amongst those responsible for Poor-Law infirmaries that no salaries should be increased during the war, but there are exceptions to every rule, as the Southwark Board of Guardians found out. At the outbreak of the war they decided that they would increase no salaries, but by an alteration in the classification of the sick poor, by which the wards of the infirmary will be relieved, additional work has been thrown upon Dr. J. C. V. Denning, the medical officer at the Christchurch institution. Here the Guardians have fitted up wards for mild chronic cases, which have now come under the care of Dr. Denning, who has some seventy more patients. It was suggested that his salary should be increased from £150 to £200 a year, but there was considerable opposition, until those who realised that with added work and responsibilities must come additional remuneration prevailed, and by a large majority the increase was granted.

### MR. HERBERT SAMUEL AND CLINICAL ASSISTANTS.

The Lambeth Board of Guardians have received a letter from the Local Government Board with reference to the engagement of clinical assistants referred to in The Hospital on October 24, page 81. The Board inquire, in the first instance, what steps the Guardians have taken to satisfy themselves that it is not at present practicable to obtain the services of fully qualified medical officers to fill the posts of those who have joined the Forces, and whether they have ascertained that the employment of clinical assistants with the duties proposed will not be in contravention of any requirements of the General Medical Council. Dr. Baly, the medical superintendent at the infirmary, in a letter addressed to the infirmary committee, states that in the past men have been obtained who have previously filled hospital appointments, and that in this way the services of the very best class of assistants have been secured, but that recently the hospital authorities were unable to supply a qualified assistant of any description. In consequence of this advertisements were issued in the usual medical papers on two separate occasions, to the first of which no reply was received, and only one to the second. He also submitted his reasons for thinking that the General Medical Council can have no objection, as their duties in fact are of a less responsible nature than those performed by the medical students in the general hospital. The Guardians have decided to forward a copy of Dr. Baly's letter to the Local Government Board, with a request that they will sanction the appointment of the clinical assistants, considering the difficulties that have arisen in procuring the services of fully qualified medical men to fill the vacant posts. We dealt with the whole question in a leading article on October 3, which we do not hesitate to recall to Mr. Herbert Samuel's recollection. In the meantime we can only repeat that the more excellent way is to offer higher salaries since the actual shortage of medical men is not a famine, and in the case of Lambeth to hand some of the district work over temporarily to private practitioners, who could attend also at the infirmary, as has been done, we understand, at the *Dreadnought* Hospital. But the first duty of the Local Government Board is to guarantee the attractions of the service.

### THE NEW KING GEORGE HOSPITAL.

One of the last acts of the King before leaving for France at the beginning of the week was to direct that the new hospital of the British Red Cross Society, which is to be located in the Stationery Offices of the Government near Waterloo Station shall be known as the "King George Hospital." This is but one more indication of the keen personal interest shown by His Majesty in hospital work, an interest which is shared by many other members of the Royal Family. Queen Mary and Queen Alexandra have both endowed beds in the new hospital, and the Queen-Mother celebrated her birthday by deciding to endow another bed, to be known as the "King Edward VII. bed." It may be noted that 1,196 beds have been obtained out of the 1,650 asked for, and when the history of the war comes to be written a prominent place should be given to the open-handed manner in which the public has met any call made upon it for alleviating the sufferings of the wounded as far as it may be humanly possible.

### MIDDLESEX HOSPITAL AND THE WAR.

Prince Alexander of Battenberg has presented an interesting report on the work of the hospital during the last six months to the court of governors of the Middlesex Hospital. It will be remembered that when Prince Alexander of Teck had to resign the chairmanship of the hospital on being appointed Governor-General of Canada, Prince Alexander of Battenberg undertook to fill his place. The latter, indeed, who has been serving with the Expeditionary Force, has set an example, and is now continuing his work for the hospital since he was invalided home, and will go on with it until he returns to the Front. The number of beds placed by the Middlesex Hospital at the disposal of the War Office and the nature of the work performed have been described lately in our columns. As to the staff, owing to all the hospital's surgeons and physicians having been commissioned in the Royal Army Medical Corps for service at the 3rd London Hospital, their place at the branch hospital at Clacton has been taken by the obstetric officers. Moreover, as Prince Alexander pointed out, the improvement schemes initiated early in the year have not been dropped, but, so far as is possible, are being carried forward.

### THE WOMEN'S VOLUNTARY HOSPITALS BRIGADE.

We have received a large number of letters from correspondents offering their services in response to the notice published in our columns last week. The majority of these correspondents seem under a misapprehension as to the character of the service which can be rendered, for they appear to believe that it is in connection with nursing the wounded, or in direct association with them. Such is not the work open to members of the Brigade. That work would be for each of its members to take in hand under officers of the League of Mercy the receipt of contributions from the small givers to voluntary hospitals during the period of the war. Membership of the League of Mercy involves the duty of raising or collecting each year not less than £1. The League of Mercy receives all such collections, and hands them over without deduction to King Edward's Hospital Fund for London and to other voluntary hospitals outside the Metropolitan area. It is believed that the Women's Voluntary Hospitals Brigade, if formed, might prove a notable unit in the forces which are fighting the battle of the sick and wounded, whose headquarters are the great voluntary hospitals. These institutions must not be allowed to suffer from lack of funds during the war, and to secure this an army of voluntary workers is needed. That is the kernel of the matter. All grounds for misapprehension being now removed, we invite every new applicant who would be willing to join the Women's Voluntary Hospitals Brigade to write to the Editor of The Hospital at once; and all our correspondents, who have already written, *to send us a postcard to confirm their previous offer, or to cancel it.* There will thus be created *a clear record that our correspondents understand exactly the nature of the work, and are prepared to enter upon it, "con amore," should their offer be accepted.*

### THE GROWING LIST OF NOTIFIABLE DISEASES: GUARDIANS' CRITICISMS.

At an inquest held recently at Walsall the local Board of Guardians was severely criticised by the Coroner because, owing to the fact that the Guardians do not possess an ambulance, one of their relieving officers had hired a taxi-cab to remove a patient suffering from erysipelas to the infirmary. In the discussion at the board one of the members animadverted upon the number of diseases now notifiable. He said: "The list would continue to grow so long as a fee attached to the giving of a notification certificate. Only recently ophthalmia neonatorum was made a notifiable disease, and many people were greatly alarmed to think they had lived so long without any attempt being made to safeguard them against such a danger." The fatuity of the criticism is only equalled by the ignorance displayed in the selection of the disease to support the argument. Notification has met with opposition from many medical men on account of the breach of professional secrecy it involves. It has been enforced because of its paramount importance to the public health. The inclusion of ophthalmia neonatorum amongst notifiable diseases is due to the fact—known to everyone interested in public health—that it is the cause of about 30 per cent. of blindness. The fees paid for its notification are repaid a thousand fold by the opportunity afforded for ensuring the provision of proper treatment and for thus reducing the total amount of blindness in the community. With increased knowledge, and as the advantages of notification become more generally realised, the list of notifiable diseases is likely to be very greatly increased, with a corresponding gain to the health of the people.

### THE SECRETARY OF THE "C.O.S." RESIGNS.

A severe loss has been experienced by the Charity Organisation Society through the resignation of that well-known social worker, Dr. C. S. Loch, who has resigned the post of secretary after holding office for more than thirty-eight years, owing to continued ill-health. Though the work of the Society is not primarily concerned with hospital questions, institutional life touches social problems at so many points, particularly through the almoner's department, that all who realise practically the complexity of the problems presented and the value of method and organisation will regret the retirement from active work of Dr. Loch, who may be described as the father of thoroughness in social work. His critics have sometimes complained of red tape and pigeon-holes being given an undue importance by the "C.O.S.," but at the time when he began his work there was need for more of both of them; and after all his main achievement is perhaps to have indicated and checked the tendency to overlapping which is always a danger in charitable concerns.

### POOR-LAW AUTHORITIES AND THE WAR.

Boards of Guardians from England and Wales were well represented at the annual meeting of the Association of Poor-Law Unions held last week. Sir John Spear, M.P., presided, and among the many subjects of interest which came up for discussion the one in which public opinion is now most concerned is the care of dependants of sailors and soldiers dying through the war. Although a resolution, framed by the Council, urging that national resources should be used for providing for these dependants was carried, the discussion showed almost as much confusion of mind as in less well-instructed circles. Mr. Elwin urged that men permanently maimed should also be remembered when provision was under discussion, but when Mr. Gibbs declared that 7s. 6d. was totally inadequate for a widow, Bumble's voice was heard crying "Oh! oh!" for all the world as if Dickens were still alive to record the fact. Again, the Rev. P. S. G. Propert declared that potential recruits were holding back from mercenary motives, as if a true patriot were less bound to honourable terms for dangerous work, terms easily within the power of the nation, than other folk. The sort of patriot who throws common sense to the winds and fights for his country while leaving his wife and child unprovided for at home can never make a first-rate soldier. And to exploit the sentiment of patriotism in order to pay men less than their due is more shameless than the most mercenary motive could ever be. In point of fact, mercenary motives never will make men fight in days when the profession of mercenary soldier has gone the way of all the feudal forms of war. Mr. Leach, of Rochdale, knew more what the nation is thinking of when he said that, if adequate provision were not made for dependants, Boards of Guardians would have to augment the pensions, and was speaking as a patriot as well as a Poor-Law officer when he retorted on an interrupter that, if the guardians were doing so now, it was a great shame that they should have to. At all events, that is what the nation feels, and the point for the Association to grasp is that, in declining to pauperise the nation in this way, except after the strongest protest, they will have public opinion behind them.

### THIS WEEK'S DRUG MARKET.

Business continues fairly good, although the high prices quoted for a large number of drugs naturally have the effect of restricting orders to much smaller dimensions than at normal times. In the case of most of the drugs which advanced in consequence of the war, the values are likely to be maintained so long as the war lasts; and while it would hardly be advisable to purchase a larger supply than is necessary to cover requirements for a little time ahead, not much advantage can be obtained by continually delaying the making of purchases. Price fluctuations during the week have not been numerous. Opium has again advanced, and still higher prices are expected; morphine and codeine are also dearer, and the upward tendency will probably be maintained. Senna leaves are fetching higher prices. Ipecacuanha root is dearer. Oil of peppermint has a lower tendency in price. Quinine is unchanged. The demand for cod-liver oil is quiet in view of the fact that the chief consuming season has begun; the price asked is low considering all the circumstances, and with a brisk demand would probably advance. Eucalyptus oil is also in small request, and prices are very moderate. Carbolic acid continues to tend upwards in price.

### TO OUR READERS.

Contributions are specially invited from any of our readers to these columns. They should deal with topical subjects and news. They must be authenticated for the information of the Editor only. The minimum payment if published is 5s. There is no hard-and-fast rule as to space, but notes of about twenty lines in length are preferred.

